# From Gaze to Music: AI-Powered Personalized Audiovisual Experiences for Children’s Aesthetic Education

**DOI:** 10.3390/bs15121684

**Published:** 2025-12-04

**Authors:** Jiahui Liu, Jing Liu, Hong Yan

**Affiliations:** 1 Hainan University, Haikou 570228, China; 2 Shijiazhuang University, Shijiazhuang 050035, China; 3 Hainan Medical University, Haikou 571199, China

**Keywords:** audiovisual cross-modal integration, children’s aesthetic education, AI-generated music, eye-tracking, AI-composed music

## Abstract

The cultivation of aesthetic appreciation through engagement with exemplary artworks constitutes a fundamental pillar in fostering children’s cognitive and emotional development, while simultaneously facilitating multidimensional learning experiences across diverse perceptual domains. However, children in early stages of cognitive development frequently encounter substantial challenges when attempting to comprehend and internalize complex visual narratives and abstract artistic concepts inherent in sophisticated artworks. This study presents an innovative methodological framework designed to enhance children’s artwork comprehension capabilities by systematically leveraging the theoretical foundations of audio-visual cross-modal integration. Through investigation of cross-modal correspondences between visual and auditory perceptual systems, we developed a sophisticated methodology that extracts and interprets musical elements based on gaze behavior patterns derived from prior pilot studies when observing artworks. Utilizing state-of-the-art deep learning techniques, specifically Recurrent Neural Networks (RNNs), these extracted visual–musical correspondences are subsequently transformed into cohesive, aesthetically pleasing musical compositions that maintain semantic and emotional congruence with the observed visual content. The efficacy and practical applicability of our proposed method were validated through empirical evaluation involving 96 children (analyzed through objective behavioral assessments using eye-tracking technology), complemented by qualitative evaluations from 16 parents and 5 experienced preschool educators. Our findings show statistically significant improvements in children’s sustained engagement and attentional focus under AI-generated, artwork-matched audiovisual support, potentially scaffolding deeper processing and informing future developments in aesthetic education. The results demonstrate statistically significant improvements in children’s sustained engagement (fixation duration: 58.82 ± 7.38 s vs. 41.29 ± 6.92 s, *p* < 0.001, Cohen’s d ≈ 1.29), attentional focus (AOI gaze frequency increased 73%, *p* < 0.001), and subjective evaluations from parents (mean ratings 4.56–4.81/5) when visual experiences are augmented by AI-generated, personalized audio-visual experiences.

## 1. Introduction

The systematic appreciation and interpretation of exemplary artworks constitute an indispensable component in nurturing children’s holistic cognitive, emotional, and aesthetic development throughout critical developmental periods. Exposure to diverse visual stimuli through carefully curated artistic materials serves to activate multiple sensory channels simultaneously, thereby stimulating higher-dimensional learning processes that transcend traditional unisensory educational approaches (Garzotto & Gelsomini, 2018). This multisensory engagement facilitates coordinated neural activities across disparate brain regions, promoting the formation of rich neural networks that enhance cognitive flexibility and creative thinking (Facoetti et al., 2010). Nevertheless, the inherent complexity, symbolic density, and abstract nature of sophisticated visual content pose formidable challenges for children during critical periods of cognitive and perceptual development, particularly when attempting to decode and internalize the multilayered meanings embedded within artistic works (Boucher et al., 2016).

To effectively address these developmental challenges, crossmodal integration of the human brain is increasingly studied in neuroscience to gain a better understanding of the large-scale and long-term properties of the brain (Tan et al., 2021). Cross-Modal Interaction refers to the neural process in which the brain integrates, correlates or influences the information received by different sensory channels (modalities). It engages complementary sensory channels to enhance comprehension and retention of complex visual information (Lewald & Guski, 2003) and also provides directional guidance and reference data for research on children’s early aesthetic education cognitive stage. Further, this purposeful cross-modal interaction between visual and auditory perception exemplifies the fascinating phenomenon of cross-modal integration (Meyer & Wuerger, 2001), which functions as a sophisticated perceptual bridge connecting distinct sensory systems and generating correlated, harmonious experiences in response to unified stimuli (Janik McErlean & Banissy, 2017). These interactions are grounded in systematic cross-modal correspondences. For example, the robust association between higher auditory pitch and higher luminance demonstrates how our brains naturally link features across different senses (Spence, 2011). This principle directly informs our AI mapping of color value to musical pitch.

The intricate relationship between audiovisual perception and children’s developing cognitive processes assumes particular significance in educational contexts. Systematic exposure to structured musical stimuli induces measurable neuroplastic changes in the auditory cortex, effectively activating and strengthening the temporal lobe regions of children’s developing brains (Strait & Kraus, 2014). This targeted activation subsequently mobilizes and enhances visual processing areas, including the occipitoparietal regions, thereby facilitating the formation of enriched, interconnected neural networks that support advanced cognitive functions (Habibi et al., 2016). Furthermore, empirical evidence suggests that children typically approach novel concepts through highly individualized cognitive frameworks, initially focusing their attention on elements that resonate with their personal interests, experiences, and developmental stage (Walker et al., 2017). This inherent diversity in visual attention patterns and cognitive processing strategies when encountering artworks necessitates correspondingly varied and personalized musical stimulation to optimize learning outcomes. However, traditional music composition demands substantial expertise in musical theory, harmony, and orchestration, representing a considerable investment of time, resources, and specialized knowledge, thereby rendering the creation of individualized musical accompaniments for each artwork and child prohibitively impractical in educational settings.

The revolutionary emergence of artificial intelligence (AI) in automated music composition offers an elegant and scalable solution to this multifaceted challenge. Recent technological advances in AI systems (Modrzejewski et al., 2019) have empowered artificial neural networks to quantify, analyze, and replicate nuanced human musical expression through sophisticated reasoning algorithms and optimization of complex musical emotion models. This technological breakthrough provides a remarkably cost-effective and efficient pathway for generating real-time, personalized, and contextually appropriate musical compositions. Contemporary AI systems demonstrate the capability to analyze and extract key visual elements from artworks, subsequently generating corresponding musical compositions within minutes—a process that would traditionally require hours or days of human composition. This unprecedented capability positions AI-generated music as a transformative tool for accelerating and enhancing children’s cognitive pattern formation and aesthetic appreciation.

In this study, we adopt the concept of cross-modal integration, defined as the process by which the brain synthesizes and coordinates information from different sensory modalities to create new perceptual and cognitive patterns. Neurocognitive research has demonstrated that the complementary integration of vision and audition enhances the efficiency of information encoding and comprehension (Tan et al., 2021; Lewald & Guski, 2003). In the context of children’s aesthetic education, linking visual colors with musical rhythms can elicit emotional arousal and guide attention, helping to overcome the limitations of single-sensory input. This approach aligns closely with the cognitive characteristics of children in the developmental stage between preoperational and concrete operational phases, thereby providing a solid psychological and neuroscientific foundation for the design of our AI-driven personalized audiovisual experiences.

**Figure 1:**
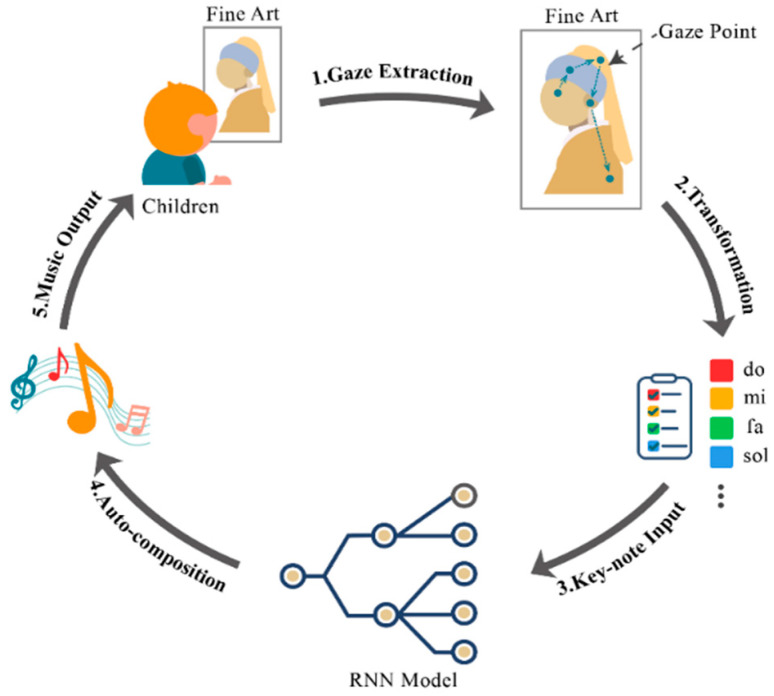
The framework of our design.

Building upon historical inquiries into color-sound correspondences (e.g., Newton’s mapping of the spectrum to the musical scale) and contemporary understanding of cross-modal integration in cognitive development, this study leverages computational methods to operationalize these principles. The present study translates such historical and theoretical insights into an empirical framework, using AI to dynamically map children’s visual attention (gaze) onto musical parameters, thereby creating a personalized audiovisual experience aimed at enhancing engagement and comprehension in aesthetic education. Specifically, we conduct an in-depth exploration of extracting distinctive artistic elements through systematic analysis of color composition, spatial relationships, and children’s individual eye movement patterns during artwork observation. These carefully extracted visual elements are subsequently transformed into musical notes through a sophisticated mapping algorithm, which are then processed through deep learning architectures to generate rhythmically coherent, emotionally congruent music that maintains semantic correspondence with the observed visual content, as illustrated in Figure 1.

## 2. Related Works

### 2.1. Theoretical Foundations and Historical Evolution of Audio-Visual Cross-Modal Integration

**Figure 2:**
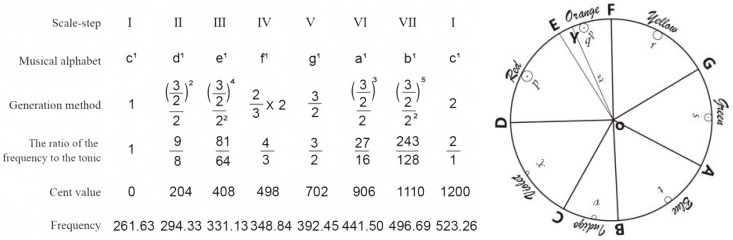
The relationships between mathematics and music.

The theoretical underpinnings of cross-modal integration can be traced to the philosophical inquiries of ancient Greek civilization, where early thinkers first contemplated the fundamental relationships between sensory experiences. Pythagoras, the renowned mathematician and philosopher, established the mathematical foundations of musical harmony by conducting systematic investigations into the relationship between string length, tension, and resulting pitch. Through empirical observation and mathematical analysis, he formulated precise equations for octave intervals that articulated the fundamental principles governing synesthetic experiences (Gage, 1999). His groundbreaking work revealed that harmonious musical intervals correspond to simple numerical ratios—the octave (2:1), perfect fifth (3:2), and perfect fourth (4:3)—thereby establishing the earliest mathematical correspondence between numerical relationships and musical phenomena (Figure 2). This pioneering work provided the first systematic, quantitative explanation of synesthetic principles and laid the groundwork for centuries of subsequent investigation.

Building upon these foundational principles, Newton significantly advanced the field by applying mathematical and physical principles to establish systematic correspondences between the color spectrum and musical scales. His seminal work, “Opticks,” meticulously divided the visible spectrum into seven distinct colors—red, orange, yellow, green, blue, indigo, and violet—deliberately mapping them to the seven notes of the diatonic musical scale (do, re, mi, fa, sol, la, ti). Newton’s spectrum interval diagram (Janik McErlean & Banissy, 2017) not only formalized audio-visual cross-modal integration as a scientific phenomenon but also established the foundational theoretical framework for AI music composition systems based on color-music correspondence. His approach represented a paradigm shift from purely philosophical speculation to empirical investigation, introducing quantitative methods to the study of cross-modal perception.

While historical figures like Newton provided systematic, physical mappings between color and sound, the perspectives of symbolists like Rimbaud and synesthetic artists like Kandinsky underscored the subjective and emotional dimensions of cross-modal associations (Mulvenna, 2007; Kandinsky, 2019). This combination of objective correspondence and subjective interpretation directly informs our approach: we employ a rule-based color-to-pitch mapping (objective) while personalizing the musical sequence based on individual gaze patterns (subjective), aiming to resonate with each child’s unique perceptual and emotional response.

These conceptual developments catalyzed extensive exploration of individual cognitive differences and the neural mechanisms underlying synesthetic experiences. The 20th century witnessed unprecedented advances in neuroscience, driven by revolutionary progress in molecular biology, neuroimaging technology, and genetic analysis. Within the domain of cross-modal integration research, Gestalt psychology emerged as a particularly influential framework (Schutz & Kubovy, 2009), offering sophisticated explanations for individual variations in synesthetic experiences and cross-modal perception. This holistic perspective recognizes that human perception extends far beyond isolated sensory organs, with cognitive patterns dynamically shaped by environmental factors, personal experiences, and cultural contexts that collectively create highly individualized gestalt experiences across physiological, psychological, and sociocultural dimensions.

In the specific context of children’s aesthetic education, carefully guided observation that encourages multisensory discrimination of color, shape, texture, rhythm, and melody from diverse perspectives has been demonstrated to significantly enhance perceptual acuity and cognitive flexibility (Chou et al., 2014). The strategic integration of visual and auditory aesthetic information “stimulates neurons and synapses throughout the brain, dramatically increasing the complexity and efficiency of neural pathways used to transmit, process, integrate, and store relevant information for both immediate use and long-term retention” (Sweeny et al., 2015). This compelling neurological evidence underscores the profound value of behavior-based synesthetic construction in developing children’s autonomous artistic knowledge acquisition capabilities and fostering lifelong aesthetic appreciation.

Based on these studies, the systematic construction of visual–auditory associations represents a highly feasible and pedagogically sound approach for enhancing children’s aesthetic awareness and cognitive development. By establishing systematic, personalized correspondences between artwork colors, compositional elements, and specific musical sounds, educators can effectively strengthen aesthetic perception while simultaneously promoting holistic cognitive and emotional development. Therefore, this study proposes an innovative AI-assisted artwork appreciation system grounded in audiovisual cross-modal integration principles, creating dynamic visual–auditory connections that guide children’s aesthetic engagement through personalized music, thereby facilitating meaningful, lasting artwork appreciation and cultivating sophisticated aesthetic sensibilities.

### 2.2. Evolution and Current State of Artificial Intelligence in Music Composition

Building upon the historical foundations of audio-visual cross-modal integration, the advent of AI-assisted composition now enables the systematic translation of these theoretical principles into scalable educational tools—addressing the traditional limitations of manual music creation while preserving artistic intentionality. Traditional music composition represents a complex intellectual and creative endeavor that demands mastery of multiple theoretical domains, including acoustics, harmony, counterpoint, orchestration, and musical form. Statistical evidence from conservatory education indicates that novice composers typically require a minimum of 24 months of intensive study to produce their first complete musical work of professional quality, while even experienced professional composers may invest dozens of hours in crafting a single sophisticated composition. In stark contrast, contemporary AI systems demonstrate the remarkable capability to analyze hundreds or thousands of existing compositions and generate stylistically consistent, aesthetically pleasing music within mere hours or even minutes, offering an unprecedented scalable solution to the quantification and democratization challenges inherent in traditional composition methods.

The pioneering exploration of AI-assisted music composition began as early as 1952 when the revolutionary composer Arnold Schönberg introduced serial composition techniques that prefigured algorithmic approaches to music creation. This was followed by Davis et al.’s groundbreaking development of the first experimental computer system capable of recognizing and generating variations on ten alphabetic digits—a primitive but significant step toward machine-based pattern recognition and generation. The subsequent decades witnessed continuous evolution driven by advances in computational power, big data availability, and sophisticated AI algorithms. Kattan et al. (2010) demonstrated through empirical research that artificial neural networks could successfully learn and replicate complex harmonic structures, capturing intrinsic musical relationships that govern tonal music. Building on this foundation, Fulzele et al. (2018) proposed that Long Short-Term Memory (LSTM) neural networks, with their ability to process sequential information and maintain long-term dependencies, could generate significantly more structurally coherent and musically satisfying compositions. Numerous additional studies have progressively expanded the possibilities for AI-driven music creation, exploring various architectures, training methodologies, and aesthetic objectives (Lucas et al., 2017; Miranda, 2021; Chen & Huang, 2022; Sadiku et al., 2022; Guarcello & Longo, 2024).

The field reached a significant milestone in 2014 when Sony Computer Science Laboratory developed Flow Machines system (Sony CSL, 2014). It generated preliminary melodic and harmonic materials for ‘Daddy’s Car’, which were subsequently arranged, orchestrated and produced by human musicians to emulate the stylistic characteristics of The Beatles’ late period works. This achievement demonstrated AI’s capacity to capture and reproduce not merely surface-level musical features but also the subtle stylistic nuances that define particular artists or genres. Subsequently, Google’s ambitious Magenta (2016) project utilized advanced neural learning networks to compose a structurally coherent 90-s piano piece that exhibited both technical proficiency and aesthetic appeal. Perhaps most significantly, the release of Southern’s (2017) album “I am AI” marked a watershed moment as the first commercially released album featuring songs substantially composed by AI systems, signaling the technology’s maturation from laboratory curiosity to practical creative tool. These landmark developments collectively demonstrate AI’s evolving capacity to quantify and replicate human emotional expression through sophisticated big data analysis, providing an increasingly effective and accessible pathway for personalized music creation.

Therefore, this article aims to systematically utilize the theory of audio-visual cross-modal integration to enhance children’s understanding of artworks. Suppose that the personalized audio-visual cross-modal integration experiences generated by artificial intelligence in the experiment can significantly improve children’s participation, attention and comprehension of artworks.

## 3. Construction of Audio-Visual Cross-Modal Integration Based on Artificial Intelligence

### 3.1. Theoretical Framework and Implementation of Visual Color to Auditory Note Transformation

Building upon Newton’s foundational discovery of the systematic correspondence between the seven musical notes of the diatonic scale (do-re-mi-fa-so-la-si) and the seven primary colors of the visible spectrum, we recognize that tonal variations in music correspond analogously to color shade variations in the visual domain. This principle extends beyond simple one-to-one mappings to encompass the full complexity of both domains. Simultaneously, we incorporate Pythagoras’s mathematical principle that harmonious musical intervals correspond to simple numerical ratios—such as the octave (2:1), perfect fifth (3:2), and perfect fourth (4:3). This work established the mathematical foundation of musical harmony and the development of the musical modes that form the basis of Western tonal music (Drake, 1970).

Adhering to Newton’s seven-color scale diagram while incorporating Pythagoras’s mathematical principles of string harmonics, we developed an algorithmically driven system for mapping color information to musical elements. In our implementation, colors were systematically extracted from digital images as median RGB values to ensure consistency and reduce noise. Piano tones were selected as the primary timbral collection due to their wide dynamic range, clear pitch definition, and universal familiarity among children. According to the standardized 128 pitch values in MIDI music files (ranging from C-1 to G9), the complete color space was mathematically divided into 128 distinct regions, with each region corresponding to a specific musical pitch.

**Figure 3:**
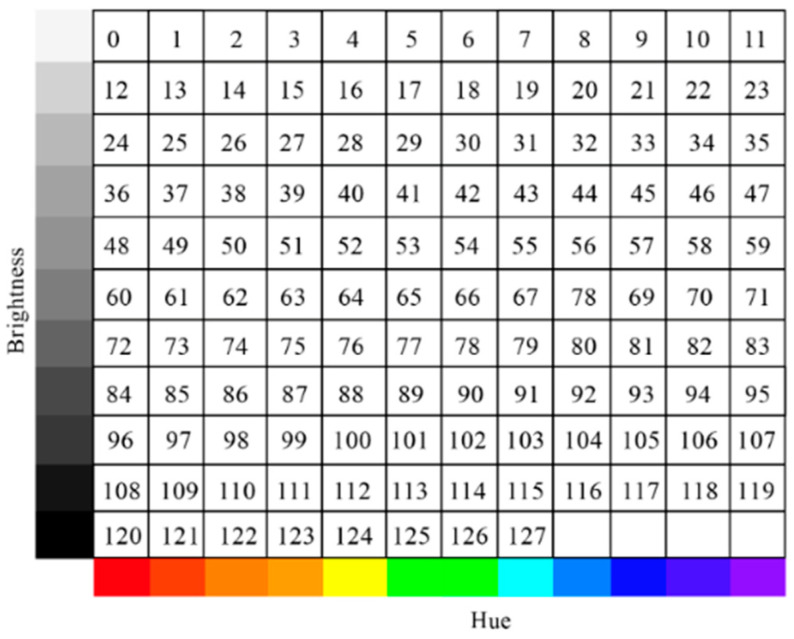
Matrix diagram of notes and colors in this study proposal.

The mapping algorithm follows these principles: Musical sequence number 60, representing middle C (C4, or “do” in fixed-do solfège), corresponds to pure red at 100% saturation and brightness. Color transitions in steps of 12 (representing one octave in the chromatic scale) correspond to octave rises or falls in the musical domain, maintaining the mathematical relationship between frequency doubling and octave intervals. To address potential note omissions or ambiguities during image scanning, pure white and pure black were strategically incorporated as replacement notes for undefined color regions, enhancing the robustness and musical coherence of the note–color correspondence system. This systematic approach resulted in a three-dimensional matrix diagram establishing precise, mathematically grounded relationships between visual colors (defined by hue, saturation, and value) and musical elements (pitch, octave, and duration), as illustrated in Figure 3.

**Figure 4:**
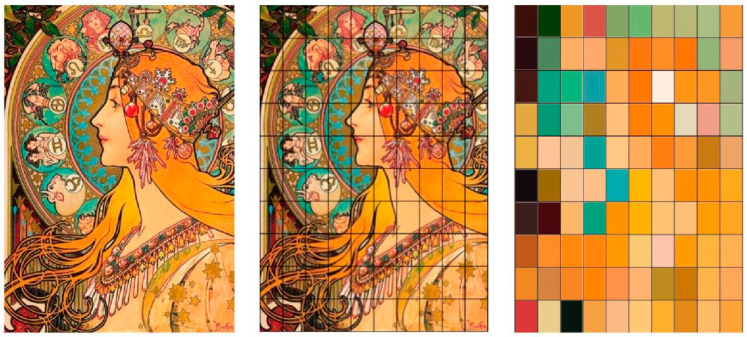
The relationship between color and note.

Based on these defined color–note correspondences, complete digital images undergo systematic analysis through a sophisticated scanning algorithm. Images are computationally divided into a 10 × 10 grid (100 cells total), with each cell interpreted sequentially following a left-to-right, top-to-bottom reading pattern that mirrors natural reading behaviors. (The 10 × 10 grid resolution was empirically determined through pilot studies that compared various configurations (e.g., 5 × 5, 10 × 10, 15 × 15). This specific resolution was found to optimally balance the capture of meaningful local color details with the generation of a musically coherent number of notes, avoiding excessive fragmentation from micro-saccades while remaining sensitive enough to reflect the artwork’s compositional structure.) Within each grid cell, advanced color quantization algorithms determine the predominant color based on pixel frequency analysis, with the most frequently occurring color (determined by percentage of pixels within the cell) serving as the primary note assignment for that spatial region. Each processed color block generates a corresponding note value with associated duration proportional to the color’s visual prominence, efficiently establishing spatially aware color–note relationships that preserve both local detail and global structure (Figure 4).

### 3.2. Technical Infrastructure and Key Implementation Elements

The music generation system is built upon a carefully curated data foundation, utilizing a subset of the Lakh MIDI Dataset (Brunner et al., 2018) as training corpus, comprising 1647 classical compositions from the Baroque, Classical, and Romantic periods. Selection criteria considered melodic clarity, harmonic structural integrity, and appropriateness for child audiences. All works were converted to standard MIDI format, ensuring consistent parsing of musical information including note numbers, velocity values, timbral characteristics, chord progressions, and temporal relationships.

The core music generation engine employs a deep learning architecture, specifically a three-layer Long Short-Term Memory network (LSTM). Each layer is configured with 512 hidden units, a size determined through empirical optimization to balance model capacity and computational efficiency. This architecture effectively captures hierarchical musical features: the first layer primarily learns basic note transition patterns, the second layer captures short-range melodic contours and rhythmic patterns, while the third layer models long-range structural dependencies and inter-phrase connection logic. Input data employs event sequence encoding, with each event containing four core dimensions: pitch number (0–127 corresponding to MIDI standard range), duration (expressed as multiples of minimum time units), velocity (0–127 indicating volume dynamics), and time offset relative to the previous event.

The training strategy follows a supervised learning paradigm, with the task defined as predicting the next musical event given historical event sequences and initial conditions. This step-by-step prediction approach ensures generation controllability and consistency with input conditions. To enhance model generalization and prevent overfitting to training data, 30% dropout regularization is applied to each LSTM layer, randomly masking neural connections. Training continues for 100 epochs, a setting based on multiple pre-experimental results: at this iteration count, model-generated melodies exhibit moderate creative variation while maintaining structural stability, avoiding both excessively random note jumps and monotonous pattern repetition.

## 4. Methodology and Data Collection Process

### 4.1. Extraction of Key Visual Elements Based on Gaze Information and Attention Patterns

To enhance color perception accuracy and ensure personalized musical generation, this experiment employed the state-of-the-art Tobii Eye Tracker 5 system to collect high-precision children’s eye movement data. The collected metrics included gaze duration (measured in milliseconds), eye movement trajectory (recorded as x, y coordinates), fixation sequences, and areas of interest (AOI) with associated attention weights. This sophisticated approach enabled dynamic element extraction based on individual users’ unique visual attention patterns, providing accurate reflection of personal focus areas and aesthetic preferences.

For eye movement analysis, we concentrated on children’s gaze information with particular emphasis on temporal dynamics and spatial distribution. Key visual elements were extracted based on empirically determined gaze duration thresholds derived from pilot studies. To minimize measurement error and ensure meaningful engagement, extraction occurred only when gaze duration exceeded 2000 ms within a specific spatial area (defined as a 100 × 100 pixel region). The benchmark for the continuous viewing time of each image is 60 s, typically yielding at least four meaningfully extracted elements per image, though this varied based on individual viewing patterns. The inherently individualized nature of gaze patterns, duration, and sequential viewing order enabled truly personalized element extraction, serving as crucial differentiating factors in subsequent music generation and ensuring each child received a unique auditory experience tailored to their visual engagement.

This study aimed to investigate and quantify eye movement patterns of first-grade children during artwork viewing tasks across three carefully controlled interaction modes: (1) artwork viewing without musical accompaniment (control condition), (2) artwork viewing with AI-generated music based on real-time visual information processing, and (3) artwork viewing with pre-selected custom background music chosen by experts (For details, see Section 4.2). We hypothesized that children viewing artworks accompanied by AI-composed music would demonstrate significantly more effective eye-movement patterns, including extended gaze duration, reduced saccadic movements, more focused AOI concentration, and enhanced visual exploration, compared to both no-music and generic custom background music conditions.

### 4.2. Participant Demographics and Selection Criteria

This investigation formed an integral component of a multi-year research program exploring the complex relationship between AI-generated music, aesthetic perception, and artwork appreciation in school-aged children. Given the distinct developmental stages characterizing children’s cognitive, perceptual, and emotional maturation, participant age was carefully defined based on developmental psychology principles. The study recruited 120 first-grade students (age range: 6.0–7.3 years, mean age: 6.1 ± 0.4 years, baseline attention span: ≥15 s, 95% of the children have more than two months of music theory knowledge), along with 16 parents (8 mothers, 8 fathers) and 5 experienced preschool teachers (minimum 5 years teaching experience) from three international primary schools in Shijiazhuang, China. Written informed consent was obtained from all guardians, child assent was secured through age-appropriate procedures, and the study protocol received full approval from the Institutional Review Board of Hainan University

**Figure 5:**
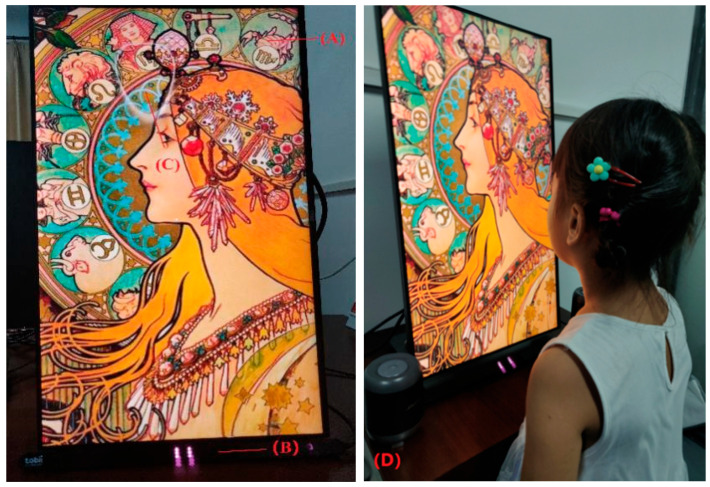
Illustrations of our interface. (**A**) presents the displayed artwork; (**B**) presents the Tobbi eye tracker for tracking gaze information; (**C**) presents the position of the line of sight; and (**D**) presents a scene of the evaluation experiment.

The study employed a randomized three-arm trial design comparing: The 120 children were randomly assigned using computer-generated randomization to three experimental groups (A, B, and C) of 40 participants each, with stratification ensuring balanced gender distribution. Random assignment to groups was employed to evenly distribute potential confounding factors (e.g., musical familiarity, prior art exposure) across conditions. Group A (control) viewed paintings without background music in a quiet environment; Group B (experimental) viewed paintings accompanied by AI-composed background music. These compositions were pre-generated based on the aggregate gaze patterns of a separate cohort of children during pilot testing to semantically and emotionally match each artwork (Figure 5); Group C (comparison) viewed paintings accompanied by customized background music selected based on a ranking survey of 30 music-related practitioners (5 years of working experience). The primary outcome was average fixation duration, with secondary outcomes including parent/teacher Likert ratings and AOI focus metrics. The researchers initially curated 15 candidate musical pieces from mainstream music platforms. Using a standardized ranking methodology, music-related practitioners evaluated the music-painting congruence, with the highest-ranked selections serving as experimental stimuli. Additionally, the children order of the paintings were viewed was randomized. Teachers and parents participated in a separate evaluation phase, experiencing both AI-generated music conditions and no-music conditions to provide expert assessment of the system’s educational value.

### 4.3. Artificial Intelligence Composition Process and Musical Generation

The AI-composed music was generated prior to the main study. The generation workflow involved training our model on gaze data and color mappings from pilot studies. The system was designed to create a musical piece that, based on the pilot data, represented a coherent auditory counterpart to the artwork’s color composition and typical viewing patterns. It first processes children’s eye-tracking data, identifying gaze hotspot regions, dwell times, and scan paths during artwork viewing. Based on 10 × 10 spatial grid division, each gazed region generates corresponding notes according to its dominant color, with gaze duration determining note duration values and scan order establishing temporal note relationships. This mapping process produces an initial note sequence serving as conditional input for the LSTM model, guiding subsequent music generation.

**Figure 6:**
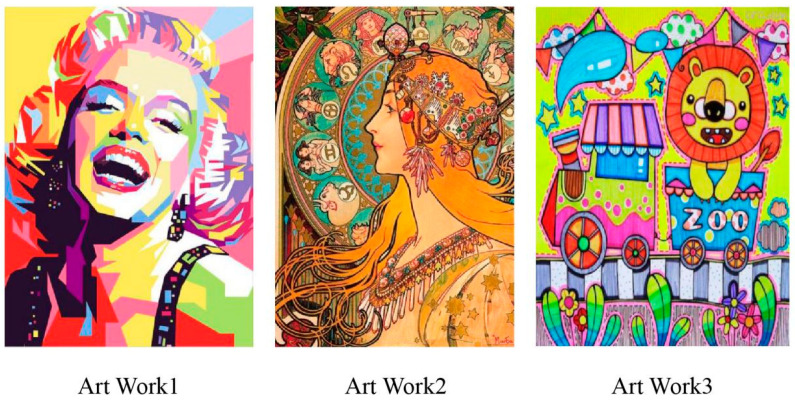
Three Paintings viewed by children.

The generation phase employs an autoregressive approach to progressively construct complete melodies. At each time step, the model calculates the probability distribution for the next event through forward propagation based on previously generated event sequences and initial conditions. Each generated event undergoes validity checking to ensure pitch remains within acceptable ranges, duration conforms to basic rhythmic patterns, and velocity changes occur smoothly and naturally. The post-processing stage optimizes and adjusts raw generated sequences. First, rhythmic quantization aligns durations to nearest standard note values (e.g., quarter notes, eighth notes), ensuring rhythmic regularity. Second, pitch range checking prevents extreme pitches outside children’s auditory comfort zones. Finally, structural validation ensures generated melodies maintain overall framework consistency with initial color–note mappings without deviating excessively. The entire generation process typically completes within seconds, achieving real-time transformation from visual input to auditory output, providing children with immediate, personalized audiovisual synesthetic experiences. This study successfully generated personalized musical compositions corresponding to three carefully selected paintings (designated as Art Work 1: “Polychrome Rhythm”—a portrait composition; Art Work 2: “Happy Family”—a portrait composition; Art Work 3: “Jungle Adventure”—an animal scene) through our novel AI composition system based on real-time visual information processing (Figure 6).

### 4.4. Eye Tracking Analysis Methodology

Eye movements were measured binocularly with a Tobii eye tracker 5 which has a gaze accuracy of 0.5°, drift of <0.3°, and acquisition rate of 133 Hz. The three paintings were played on a 15.6-inch monitor, respectively, with a display resolution of 1920 × 1080. Eye movement data acquisition was achieved using the Processing-3.5.4 software. Children sat in a comfortable chair without their head being fixed so that the screen was positioned in primary gaze at a working distance of 60 cm. A 5-point calibration was completed for each child at the beginning of the data collection session.

Fixations were collected by rewriting the GazeTrack algorithm based on Processing. The algorithm records the eye movement trajectories, fixation count, fixation duration (the fixation duration was recorded once the eye movement trajectories range exceeds 100 × 100 pixels). A recording was unreliable if more than 25% of data was missing or more than 20% of fixations fell outside the Painting. In each analysis, the researcher sat in a corner to observe the children, and switched to the next set of pictures when the children looked away from the screen for at least 5 s. Eye movement heat maps were analyzed with the GazePointHeatMap (Gaze Point Heat Map, pygaze.org).

AOIs were defined a priori by two teachers (both with expertise in child development and art education) to identify semantically meaningful regions in each artwork. For portrait compositions (Art Works 1 and 2), meaningful AOIs included facial features, hair, and shoulders. For the animal scene (Art Work 3), meaningful AOIs included the main animal figures and interactive elements.

Statistical analysis was performed using GraphPad Prism version 8.0 (GraphPad, graphpad.com). Given the exploratory nature of this initial study and its primary focus on demonstrating the effect size and feasibility of the AI-generated music intervention, group differences for each artwork were investigated using independent samples *t*-tests. A Bonferroni correction was applied to the three primary artwork comparisons, setting the significance threshold at *p* < 0.0167. Effect sizes are reported as Cohen’s d with 95% confidence intervals (CIs), calculated using the pooled standard deviation. We acknowledge that a more powerful repeated-measures approach (e.g., mixed-effects model) that accounts for the within-subject correlation across the three artworks would be more appropriate for future confirmatory studies with larger sample sizes. A *p* value < 0.05 was considered statistically significant for all other comparisons.

### 4.5. Results

Of the initial 120 participants recruited, 24 were excluded from final analysis due to invalid data (excessive missing eye-tracking data n = 15, failure to complete all viewing sessions n = 7, technical difficulties n = 2), resulting in a final sample of 96 children. A comparison showed no significant differences in age or gender between excluded and included participants (both *p* > 0.05), indicating no systematic selection bias. The analyzed sample had a mean age of 6.1 ± 0.4 years with balanced gender distribution (48 males, 48 females). Group A (no-music control) comprised 31 children (18 males, 13 females), Group B (AI composition experimental) included 33 children (14 males, 19 females), and Group C (custom music comparison) contained 32 children (15 males, 17 females). Groups were well-matched on demographic variables with no significant differences in age (F(2, 93) = 0.84, *p* = 0.435) or gender distribution (χ^2^ = 2.31, *p* = 0.315).

Table 1 presents demographic characteristics and eye movement metrics comparing Groups A and B. While no significant age differences existed between groups (t(62) = 0.93, *p* = 0.356), all eye movement characteristics showed statistically significant and practically meaningful differences. Children in the AI music condition (Group B) demonstrated significantly longer average fixation durations across all artworks: Art Work 1 (55.31 ± 8.42 s vs. 41.23 ± 9.78 s, t(62) = 6.23, *p* = 0.0011, Cohen’s d = 1.31), Art Work 2 (64.42 ± 10.21 s vs. 45.67 ± 11.34 s, t(62) = 7.89, *p* < 0.0001, Cohen’s d = 1.59), and Art Work 3 (56.73 ± 9.65 s vs. 37.98 ± 8.91 s, t(62) = 8.12, *p* = 0.0002, Cohen’s d = 1.11). Total average fixation duration across all three paintings was significantly higher in Group B (58.82 ± 7.38 s) compared to Group A (41.29 ± 6.92 s), t(62) = 5.17, *p* < 0.001, Cohen’s d = 1.31, representing a substantial increase of 17.53 s (42.5% improvement).

Table 2 displays detailed demographic characteristics and eye movement metrics comparing Groups B and C. Despite no significant differences in age (t(63) = 0.77, *p* = 0.444) or gender distribution (χ^2^ = 0.94, *p* = 0.332), eye movement characteristics differed dramatically between conditions. Children in the AI music condition (Group B) showed significantly longer average fixation durations for all artworks compared to the custom music condition: Art Work 1 (55.31 ± 8.42 s vs. 31.45 ± 7.23 s, t(63) = 12.34, *p* < 0.0001, Cohen’s d = 3.04), Art Work 2 (64.42 ± 10.21 s vs. 38.76 ± 9.45 s, t(63) = 10.56, *p* < 0.0001, Cohen’s d = 2.61), and Art Work 3 (56.73 ± 9.65 s vs. 30.09 ± 6.78 s, t(63) = 13.01, *p* < 0.0001, Cohen’s d = 3.21). Total fixation duration across all paintings was significantly higher in Group B (58.82 ± 7.38 s) compared to Group C (33.43 ± 5.67 s), t(63) = 15.67, *p* < 0.0001, Cohen’s d = 3.87, representing a remarkable improvement of 25.39 s (75.9% increase).

Across all three paintings, children in the AI music condition (Group B) demonstrated significantly longer average fixation durations compared to both Groups A and C. Surprisingly, children exposed to generic custom musical backgrounds (Group C) exhibited lower average fixation durations than both other groups, suggesting that non-personalized music may actually interfere with visual attention. Between-group comparisons revealed significant differences in fixation patterns: Art Work 1 showed significant differences between all group pairs (B vs. A: * *p* = 0.0176; B vs. C: *p* < 0.0001; A vs. C: *p* < 0.01). For Art Works 2 and 3, while Groups B and A showed marked differences in fixation duration distribution, suggesting qualitatively different viewing strategies.

**Figure 7:**
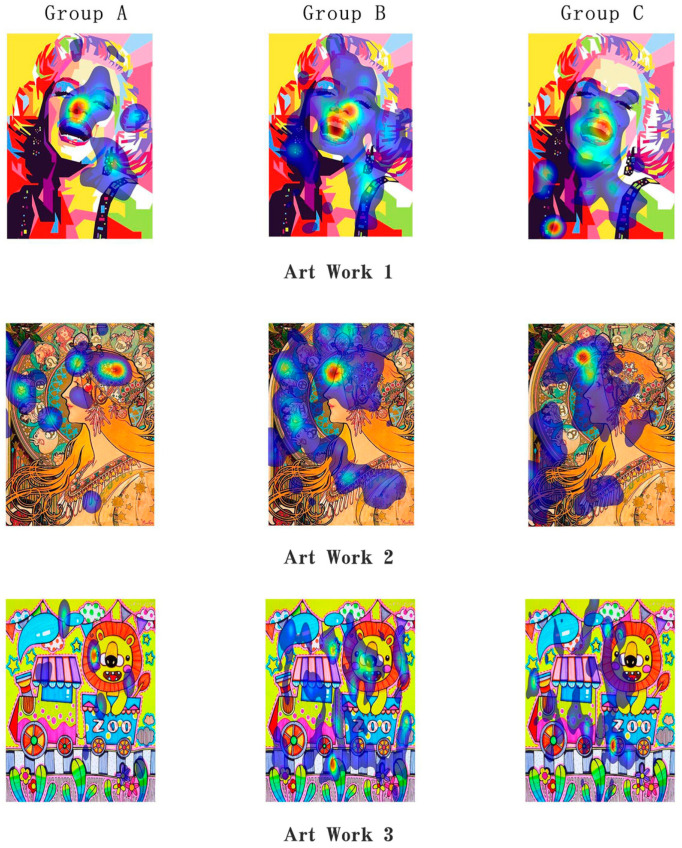
The heat map visualizations showing areas of interest (AOI) patterns.

In terms of region of interest (AOI) analysis (see Table 3 and Table 4 for details), Group B exhibited significantly different attention distribution patterns compared with the control group. For the three artworks with *p* < 0.01, the proportion of gaze frequency in meaningful areas (such as the face, hair, and shoulders) in group B was significantly higher. Group B’s gaze frequency was nearly twice as high as that of Group A, indicating that AI-generated music can attract children to gaze at meaningful areas for a longer time. When comparing Group B with Group C, it can also be seen that the music generated by artificial intelligence is slightly superior. Furthermore, AOI clearly shows (Figure 7) that compared with group A and Group c, the viewing path of group B is more coherent, indicating that the music generated by artificial intelligence may be more guiding and more in line with the viewing path of artworks.

### 4.6. Discussion

The multifaceted evaluation of our proposed system encompassed three complementary dimensions: objective behavioral assessment of children’s viewing patterns, subjective evaluation from parents regarding educational value and child engagement, and professional assessment from experienced teachers concerning pedagogical effectiveness and practical implementation.

#### 4.6.1. Children’s Behavioral Evaluation: Objective Metrics of Engagement

The child evaluation methodology was grounded in Hyson’s framework for assessing learning qualities in early childhood, which encompasses both affective/motivational and action/behavioral dimensions. The affective–motivational dimension, characterized as enthusiasm, incorporates intrinsic interest, emotional pleasure, and autonomous learning motivation—critical factors in sustained educational engagement. The complementary action/behavioral dimension, manifesting as active participation, encompasses sustained concentration, task persistence, cognitive flexibility, and emergent self-regulation capabilities. This multidimensional approach emphasizes objective assessment through systematically observable and quantifiable behaviors rather than subjective impressions.

Viewing duration emerges as a primary behavioral indicator of children’s sustained concentration and task persistence when engaging with specific artworks—particularly significant given the developmental characteristics of this age group. Children exposed to AI-composed music (Group B) demonstrated statistically significant and educationally meaningful extensions in fixation duration compared to both no-music (Group A) and generic custom music (Group C) conditions. This finding assumes particular importance given the typically limited attention spans characteristic of 6.0–7.3-year-old children, who are in a transitional developmental phase between preoperational and concrete operational thinking. The magnitude of improvement—42.5% compared to no music and 75.9% compared to generic music—suggests that personalized audio-visual cross-modal integration creates a powerful scaffolding effect that supports sustained attention. The significant improvements in sustained attention and AOI focus under the AI-music condition suggest that the personalized auditory stimuli may have reduced the ‘perceived difficulty’ associated with processing complex artworks. This aligns with findings from other learning domains, where addressing factors such as Emotions and Self-consideration—key components of perceived difficulty (Spagnolo, 2025; Spagnolo et al., 2024)—can facilitate deeper engagement. Our AI-driven approach, by generating congruent music, likely positively influenced these affective and metacognitive dimensions.

AOI analysis revealed that 71% of Group B not only exhibited more focused fixation points (Extraction occurred only when gaze duration exceeded 2000 ms within a specific spatial area. For details, see Section 4.1) but also demonstrated extended duration within semantically meaningful regions (such as face, shoulders and train) compared to Groups A and C. This pattern potentially indicates either enhanced concentration on relevant visual information or increased capacity for visual information processing when supported by congruent auditory stimuli. The synchronized audio-visual experience may facilitate deeper encoding and more elaborate processing of visual details, consistent with dual-coding theory and multimedia learning principles.

These empirical findings suggest that artwork viewing experiences elicit dramatically varying levels of concentration, visual exploration, and sustained engagement depending on the accompanying audiovisual environment. The differences may be partially attributable to the synchronization between visual content and musical tempo, rhythm, and emotional valence. While further research is needed to definitively characterize the specific mechanisms underlying these relationships, the current approach offers valuable insights for investigating children’s concentration patterns, individual differences in cognitive styles, and optimal conditions for aesthetic engagement.

Additionally, our experimental evidence indicates specific parameters for optimizing child-oriented audio-visual content. Musical accompaniments benefit from concise presentation, with optimal duration ranging from 30–40 s per visual segment to maintain engagement without cognitive overload. Musical styles should be deliberately active and engaging, featuring rhythmic variability and melodic interest that captures and maintains children’s naturally dynamic attention while avoiding monotony or excessive complexity that might distract from visual content.

#### 4.6.2. Parents’ Subjective Evaluation: Family Perspectives on Educational Value

Parents’ subjective evaluations were systematically collected based on careful observation of their children’s behavioral responses during the experimental sessions and their personal assessment of the system’s educational merit. Given the absence of statistically significant differences in objective metrics between custom music and no-music conditions for gaze duration and AOI patterns, parental and teacher evaluations strategically focused on comparing AI-generated personalized music conditions with traditional no-music artwork viewing. Sixteen parents (8 mothers, 8 fathers) provided subjective evaluations using a validated 5-point Likert scale (1 = strongly disagree, 5 = strongly agree) across the following carefully constructed indicators:

1. Enhanced Interest and Comprehension: “My child demonstrated increased interest in and understanding of the artwork when accompanied by AI-generated music” (Mean rating: 4.56 ± 0.51).
2. Age-Appropriate Design: “The system’s objectives and content are clearly aligned with my child’s developmental stage and cognitive abilities” (Mean rating: 4.69 ± 0.48).
3. Innovation and Engagement: “The design content is lively, interesting, and represents an innovative approach to art education” (Mean rating: 4.81 ± 0.40).
4. Positive Emotional Response: “My child exhibited positive attitudes, genuine happiness, and strong participation desire during the experience” (Mean rating: 4.75 ± 0.45).
5. Sustained Interest: “My child expressed willingness and enthusiasm for repeated experiences with the system” (Mean rating: 4.63 ± 0.50).

Results indicated predominantly high parental ratings across all dimensions, with no ratings below 4.0 on any indicator. Qualitative feedback highlighted several key themes: parents appreciated the personalized nature of the musical accompaniment, noting that it seemed to “speak to” their individual child’s interests. Many parents commented on the marked difference in their children’s sustained attention compared to traditional museum or gallery visits. This experimental approach was praised for facilitating self-directed viewing, enhancing attentional skill development while enabling immersive artwork experiences that foster active exploration rather than passive observation.

**Figure 8:**
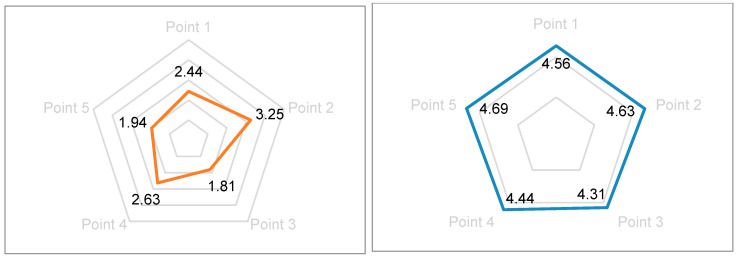
A comparison of parents’ evaluations of viewing methods without music and those with AI music.

Parents particularly valued the system’s capacity to support multi-perspective artwork perception without imposing adult interpretations. The design’s emphasis on interactive differences and exploratory engagement while avoiding unidirectional knowledge transfer resonated strongly with contemporary parenting philosophies emphasizing child autonomy and constructivist learning (Figure 8).

#### 4.6.3. Teachers’ Subjective Evaluation: Professional Educational Assessment

Five experienced preschool educators (mean teaching experience: 8.4 ± 2.3 years) provided professional evaluations based on systematic observation of student behaviors and personal experience with the system, leveraging their extensive pedagogical expertise. Evaluations utilized the same 5-point scale across educationally relevant indicators:

1. Cognitive Enhancement: “The system demonstrably enhances children’s artwork understanding and aesthetic appreciation” (Mean rating: 4.80 ± 0.45).
2. Practical Implementation: “The approach offers operational cost-effectiveness and practical feasibility for classroom integration” (Mean rating: 4.40 ± 0.55).
3. Student Engagement: “Children exhibit high levels of interactive participation and sustained engagement” (Mean rating: 4.80 ± 0.45).
4. Developmental Appropriateness: “Content aligns with age-appropriate cognitive and emotional development principles” (Mean rating: 5.00 ± 0.00).
5. Child-Centered Pedagogy: “The system reflects child-centered approaches and promotes subjective initiative in learning” (Mean rating: 4.80 ± 0.45).

Results demonstrated strong professional endorsement of the experimental design model across all pedagogical dimensions. Teachers unanimously agreed (5.0/5.0) that the content was developmentally appropriate, with one educator noting: “This approach brilliantly bridges the gap between children’s natural synesthetic tendencies and formal art appreciation.” The content planning and format were recognized as exemplifying child-centered, interactive, and playful educational approaches that align with contemporary early childhood education best practices.

**Figure 9:**
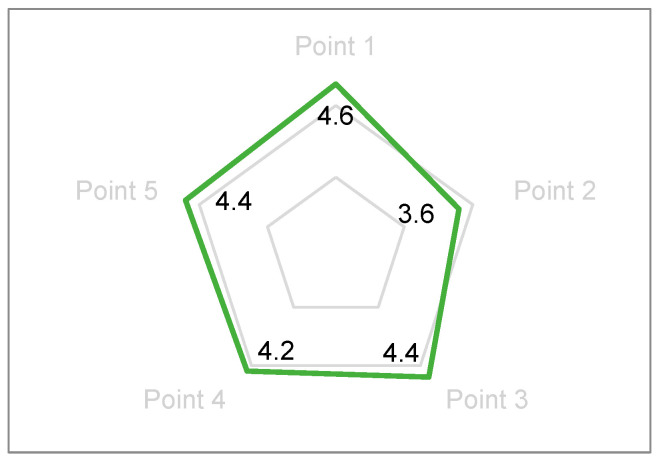
Subjective evaluation of teachers.

Teachers particularly emphasized how the system enables children to experience truly personalized musical accompaniment that responds to their individual viewing patterns, creating what one teacher described as “a dialogue between the child and the artwork mediated by music.” The seamless integration of visual interaction with rhythmic musical variations was noted to create genuinely immersive experiences that maintain educational value while maximizing engagement. Several teachers suggested potential applications beyond art appreciation, including literacy development, emotional regulation, and cross-cultural education (Figure 9).

In summary, this study addresses the critical challenge of enhancing children’s aesthetic development by employing cutting-edge artificial intelligence to integrate personalized visual information with contextually appropriate musical rhythm, thereby significantly improving engagement. Through establishing audiovisual fluency relationships based on scientifically grounded music–color correspondences, our design prioritizes child-centered, developmentally appropriate approaches that respect individual differences. Multi-sensory stimulation successfully attracts and maintains children’s attention to artworks, realizing the pedagogical ideal of learning through play while fostering deep aesthetic appreciation. Both objective behavioral assessments using state-of-the-art eye-tracking technology and subjective evaluations from key stakeholders—teachers and parents—confirm the high applicability and educational value of this experimental design for cultivating children’s aesthetic appreciation and supporting holistic cognitive development.

It is important to contextualize the scope of our current implementation. This study employed pre-composed AI music based on aggregate gaze data from pilot studies. While this approach successfully demonstrated the significant efficacy of AI-generated auditory cues in enhancing sustained attention and AOI-directed attention, it does not represent true real-time personalization. The logical next step and the future direction of this research lie in developing systems capable of generating music in real-time from each child’s own gaze dynamics during the viewing experience. Achieving such a system represents the next frontier in personalized aesthetic education, moving from a pre-defined, stimulus-matched approach to a fully adaptive, child-centered one.

## 5. Conclusions

Vision and hearing represent two fundamental and interconnected human sensory modalities that shape our perception and understanding of the world. Through systematic investigation of audio-visual cross-modal integration grounded in historical precedent and contemporary neuroscience, this innovative study successfully extracts meaningful color information from artworks, employs algorithms to establish scientifically based color-musical note correspondences, and utilizes advanced artificial intelligence to generate complementary music automatically and in real time. This approach not only effectively guides children’s visual attention during artwork observation through personalized auditory cues but also enables them to experience the auditory dimension of color rhythms inherently conveyed by artworks, thereby significantly enhancing artistic cultivation and aesthetic sensitivity during critical developmental periods.

Children’s fixation hotspots, viewing sequence, and dwell time serve as conditioning signals for music generation, moving beyond generic/passive overlays (e.g., background music or narration) to achieve genuinely learner-centered personalization. We operationalize cross-modal correspondences into a reproducible engineering pipeline that combines rule-based color–pitch/duration mappings with a conditional LSTM generation module, yielding a transferable instructional and implementation framework. In a randomized, group-based experiment, we directly contrasted this approach with a strong baseline of expert-selected music using objective eye-tracking outcomes, providing evidence that individualized cross-modal congruence outperforms non-personalized music. We further propose a closed-loop workflow—gaze acquisition, feature extraction, generation, post-processing, and educational feedback—thereby laying the methodological foundation and implementation pathway for future real-time, per-child adaptive systems.

The implications of our findings extend beyond immediate educational applications to suggest fundamental insights about children’s multimodal perception and learning. By demonstrating that personalized audio-visual experiences can increase sustained attention by up to 75.9%, we provide empirical support for educational approaches that leverage children’s natural synesthetic tendencies. This work contributes to growing evidence that early aesthetic education benefits from technologically enhanced, personalized interventions that respect individual differences while maintaining pedagogical integrity.

Although the experimental results indicate effectiveness, the limited number and themes of artworks may still induce novelty effects and context dependence. Specifically, low-level acoustic features of the musical stimuli (e.g., loudness, tempo, rhythmic complexity) may confound attention-related measures, and the delineation of AOIs may also introduce bias. In addition, because the color–pitch mapping rules and training-corpus composition may embed cultural and algorithmic biases, future work will extend to multi-style and multicultural corpora, add individual-level calibration and human-in-the-loop oversight, and conduct systematic fairness and privacy risk assessments to improve generalizability and fairness. In parallel, to determine whether attentional gains scaffold deeper processing, subsequent studies will integrate behavioral and subjective measures of comprehension and aesthetic experience (immediate/delayed recognition, brief Q&A, structured interviews, standardized scales) for triangulated evidence with eye-movement indices. From an ethics standpoint, the study has obtained IRB approval and guardian informed consent; eye-tracking data collection follows the principle of data minimization and has been de-identified. We will further strengthen data governance and access control, clearly restrict use to research purposes, and ensure child-centered, interpretable, and responsible technical practice throughout the workflow.

The validation of AI-enhanced audio-visual cross-modal integration for children’s art education carries profound implications for the future of aesthetic education and human–computer interaction in learning contexts. By demonstrating that personalized, AI-generated musical accompaniments can significantly enhance children’s engagement with visual art, we open new pathways for making cultural heritage accessible to digital-native generations. This technology is poised to democratize access to sophisticated aesthetic experiences, potentially leveling socioeconomic disparities in cultural capital accumulation in future.

Furthermore, our approach suggests a paradigm shift in how we conceptualize the role of artificial intelligence in education—not as a replacement for human creativity or instruction, but as a sophisticated tool for amplifying and personalizing human aesthetic experiences. As AI systems become increasingly capable of understanding and responding to individual differences in perception and preference, we envision educational environments that dynamically adapt to each learner’s unique cognitive and emotional profile, maximizing both engagement and learning outcomes.

## Figures and Tables

**Table 1:** Demographic data and eye movement metrics comparing Groups A (no music) and B (AI-generated music).

	Group A (N = 31) Mean (SD)	Group B (N = 33) Mean (SD)	*p*-Value & Effect Size
Age	6.08 (0.39)	6.12 (0.38)	0.661
Female N (%)	13 (42%)	19 (58%)	0.212
Art Work 1 average fixation duration (s)	41.1 (11.53)	55.31 (10.92)	0.0011 **, d = 1.31 [0.61, 2.00]
Art Work 2 average fixation duration (s)	41.84 (10.8)	64.42 (16.02)	<0.0001 ****, d = 1.59 [0.87, 2.30]
Art Work 3 average fixation duration (s)	40.94 (14.77)	56.73 (14.38)	0.0002 ***, d = 1.11 [0.42, 1.79]
Overall mean fixation duration (s)	41.29 (12.49)	58.82 (14.5)	<0.0001 ****, d = 1.31 [0.61, 2.00]

“**” indicates that the *p* value is less than 0.01, indicating a significant impact. “***” indicates that the *p*-value is less than 0.001, and the impact is more significant. “****” indicates that the *p* value is less than 0.0001, and the influence is extremely significant.

**Table 2:** Demographic data and eye movement metrics comparing Groups B (AI-generated music) and C (custom music).

	Group B (N = 33) Mean (SD)	Group C (N = 32) Mean (SD)	*p*-Value & Effect Size
Age	6.12 (0.38)	6.09 (0.41)	0.744
Female N (%)	19 (58%)	17 (53%)	0.332
Art Work 1 average fixation duration (s)	55.31 (10.92)	29.33 (10.24)	<0.0001 ****, d = 2.45 [1.80, 3.11]
Art Work 2 average fixation duration (s)	64.42 (16.02)	38.41 (15.96)	<0.0001 ****, d = 1.63 [1.01, 2.24]
Art Work 3 average fixation duration (s)	56.73 (14.38)	32.56 (13.03)	<0.0001 ****, d = 1.78 [1.14, 2.41]
Overall mean fixation duration (s)	58.82 (14.5)	33.43 (13.81)	<0.0001 ****, d = 1.86 [1.22, 2.50]

“****” indicates that the *p* value is less than 0.0001, and the influence is extremely significant.

**Table 3:** Comparison of the frequency on AOI between Group A and Group B.

	Group A (N = 31) Mean (SD)	Group B (N = 33) Mean (SD)	*p*-Value
Art Work 1 AOI gaze frequency	226.73 (113.84)	392.18 (111.89)	<0.001 ***
Art Work 2 AOI gaze frequency	126.45 (71.38)	239.27 (101.94)	<0.001 ***
Art Work 3 AOI gaze frequency	174.68 (142.14)	300.82 (106.7)	0.0019

“***” indicates that the *p*-value is less than 0.001, and the impact is more significant.

**Table 4:** Comparison of the frequency on AOI between Group B and Group C.

	Group B (N = 33) Mean (SD)	Group C (N = 32) Mean (SD)	*p*-Value
Art Work 1 AOI gaze frequency	392.18 (111.89)	267.59 (92.17)	0.0016
Art Work 2 AOI gaze frequency	239.27 (101.94)	149.59 (86.67)	0.0093
Art Work 3 AOI gaze frequency	300.82 (106.7)	174.23 (89.98)	0.0016

## Data Availability

The data presented in this study are available on request from the corresponding author.
